# Protamine-Exacerbated Right Ventricular Failure After TAVR in Critical Aortic Stenosis

**DOI:** 10.1016/j.jaccas.2026.109339

**Published:** 2026-07-29

**Authors:** Jaideep Menda, Jiun-Ruey Hu, Christy Slingwine, Sabah Skaf, Meshe Chonde, Wen Cheng, Suhail Dohad, Raj R. Makkar, Tarun Chakravarty

**Affiliations:** a Smidt Heart Institute, Cedars-Sinai Medical Center, Los Angeles, California, USA; b Department of Anesthesiology, Cedars-Sinai Medical Center, Los Angeles, California, USA

**Keywords:** protamine, right ventricle, structural intervention, transcatheter aortic valve replacement

## Abstract

**Background:**

Protamine is routinely administered for heparin reversal after transcatheter aortic valve replacement (TAVR), but protamine reaction is an under-recognized and potentially catastrophic complication.

**Case Summary:**

A 79-year-old man with critical aortic stenosis, baseline severe right ventricular (RV) dysfunction, and 2-vessel coronary artery disease underwent TAVR. Immediately following protamine administration for heparin reversal, he developed abrupt hemodynamic collapse with acute RV pressure overload on echocardiography requiring cardiopulmonary resuscitation. Despite inhaled nitric oxide, inotropes, and intra-aortic balloon pump, he remained hypotensive. Insertion of Impella RP resulted in immediate improvement in hemodynamics, with drop in central venous pressure from 31 to 20 mm Hg and improvement in oxygenation and RV contractility.

**Discussion:**

Early initiation of RV support with Impella RP should be considered in patients with acute RV failure secondary to protamine reaction.

**Take-Home Message:**

Patients with preexisting RV dysfunction may be susceptible to catastrophic type III protamine reaction because of limited physiologic reserve.

## Visual Summary

**Figure:**
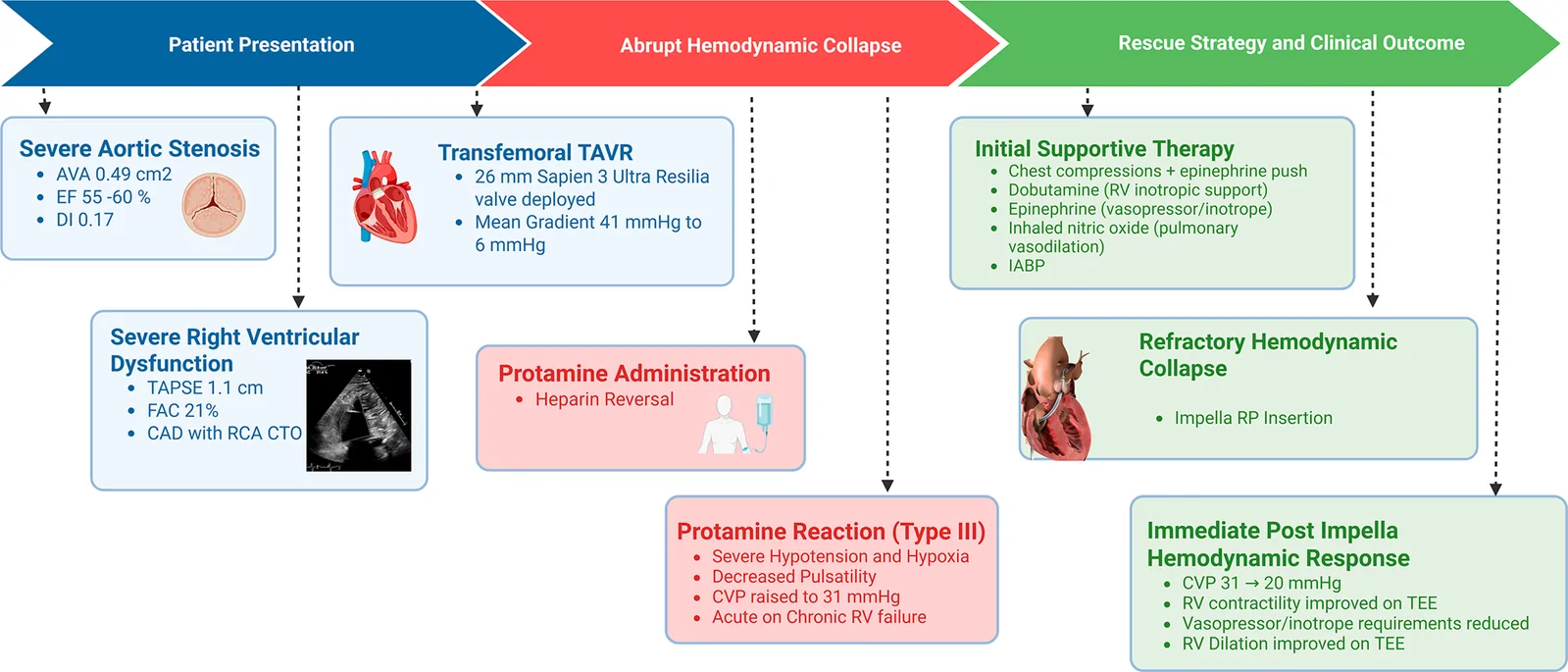

**Visual Summary:**
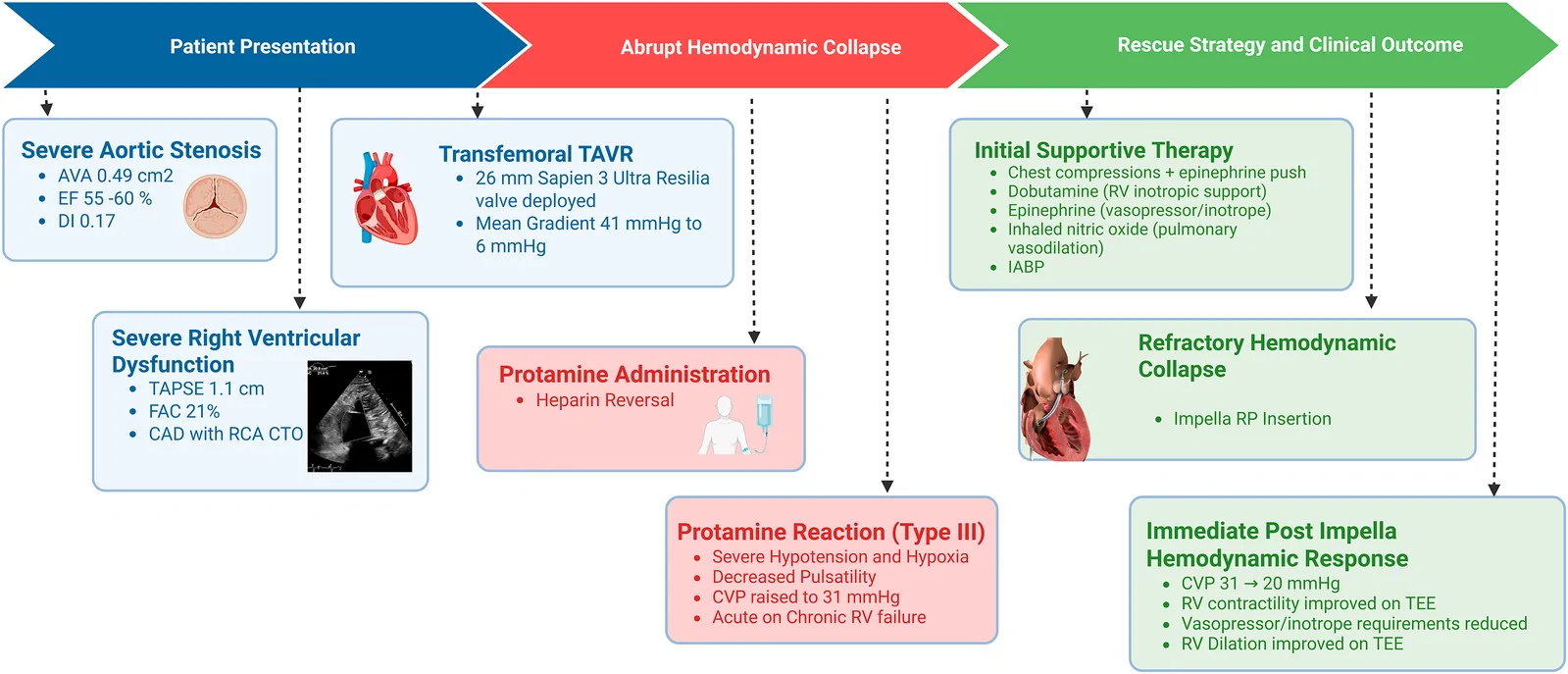
Timeline of Protamine-Induced RV Failure After TAVR AVA = aortic valve area; CAD = coronary artery disease; CTO = chronic total occlusion; CVP = central venous pressure; DI = dimensionless index; EF = ejection fraction; FAC = fractional area change; IABP = intra-aortic balloon pump; RCA = right coronary artery; RV = right ventricular; TAPSE = tricuspid annular plane systolic excursion; TAVR = transcatheter aortic valve replacement; TEE = transesophageal echocardiography.

## History of Presentation

**Figure 1:**
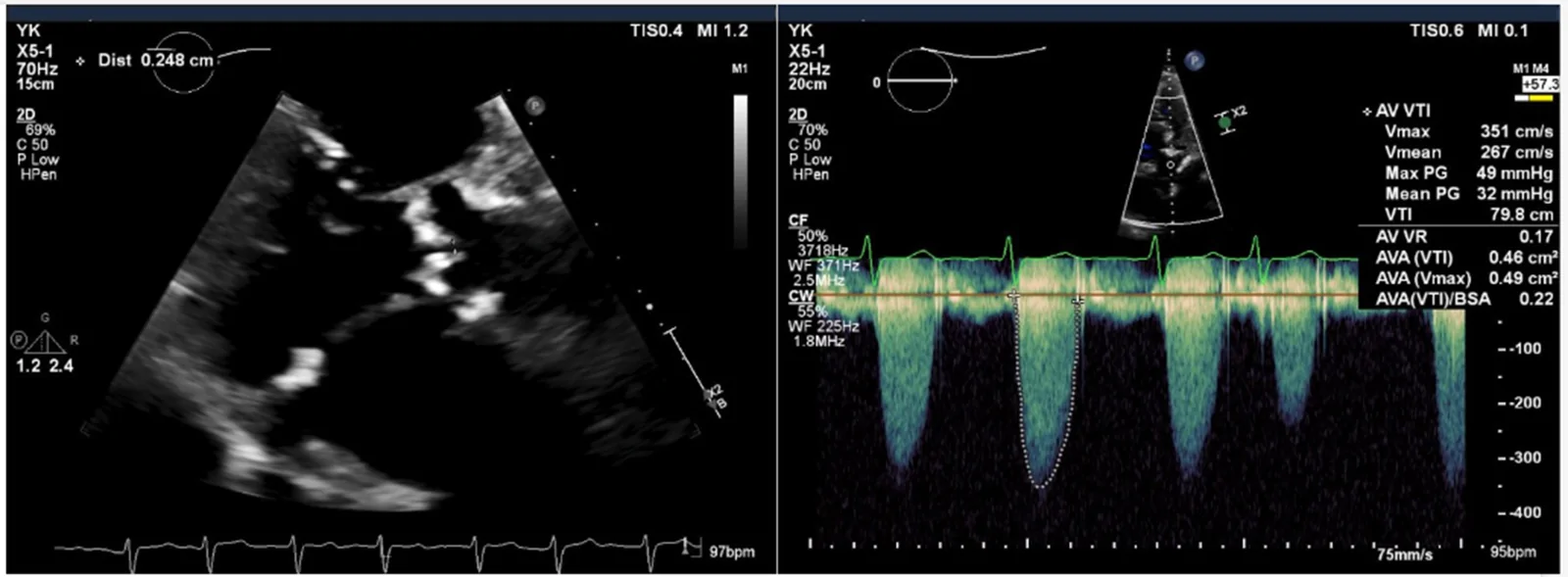
Transthoracic Echocardiography on Presentation Showing Aortic Stenosis (Left) Aortic-focused zoomed-in parasternal long-axis view demonstrating severely reduced aortic valve cusp separation (0.25 cm). (Right) Continuous-wave Doppler in the apical 4-chamber view demonstrating aortic valve area of 0.49 cm^2^, suggesting critical aortic stenosis, with a mean gradient of 32 mm Hg, and maximum velocity of 3.51 m/s.

**Figure 2:**
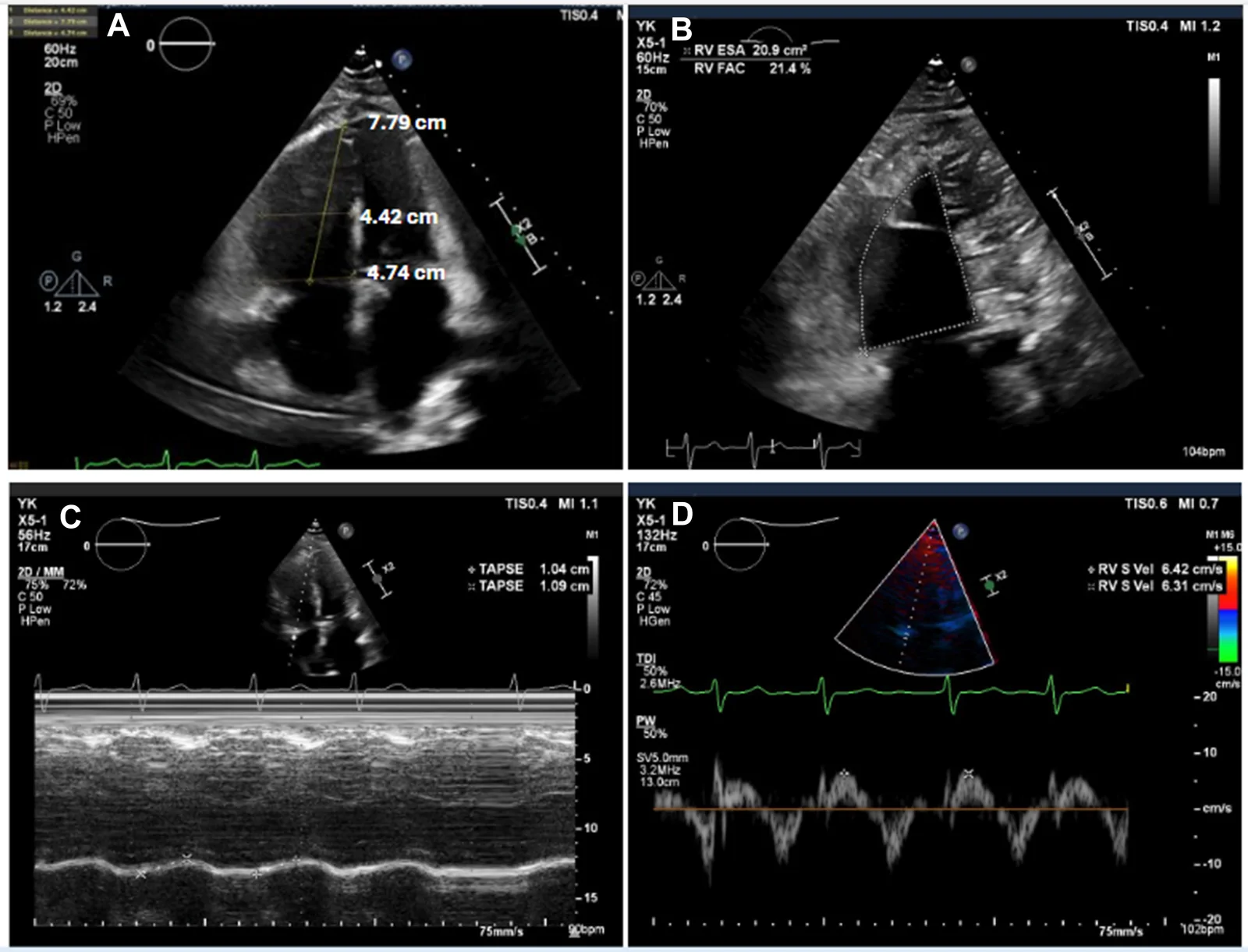
Right Ventricular Dysfunction at Baseline (A) RV-focused apical 4-chamber view on TTE demonstrating dilated right ventricle, with a mid-RV short-axis width of 4.42 cm and basal RV short-axis width of 4.74 cm. (B) RV-focused apical 4-chamber view on TTE demonstrating fractional area change of 21.4%, consistent with dilated RV. (C) TAPSE in M-mode is reduced at 1.04 cm, consistent with reduced RV systolic function. (D) Pulse-wave Doppler of the RV septum on TTE demonstrating reduced RV S′ of 6.31 cm/s, consistent with reduced RV systolic function. RV = right ventricular; TAPSE = tricuspid annular plane systolic excursion; TTE = transthoracic echocardiography.

**Figure 3:**
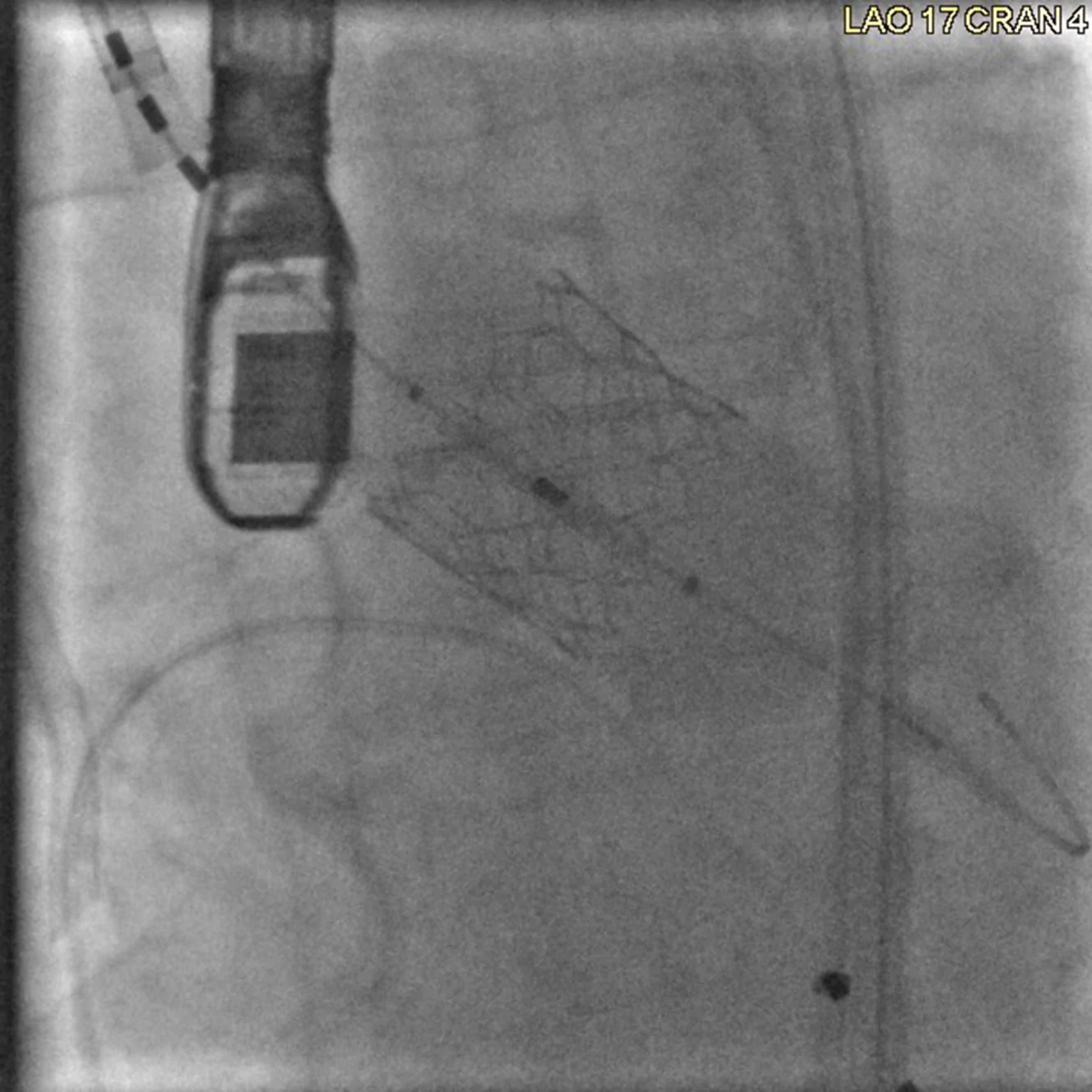
Fluoroscopy Demonstrating Deployment of the 26-mm Sapien 3 Ultra Resilia Bioprosthetic Valve Under Rapid Pacing

**Figure 4:**
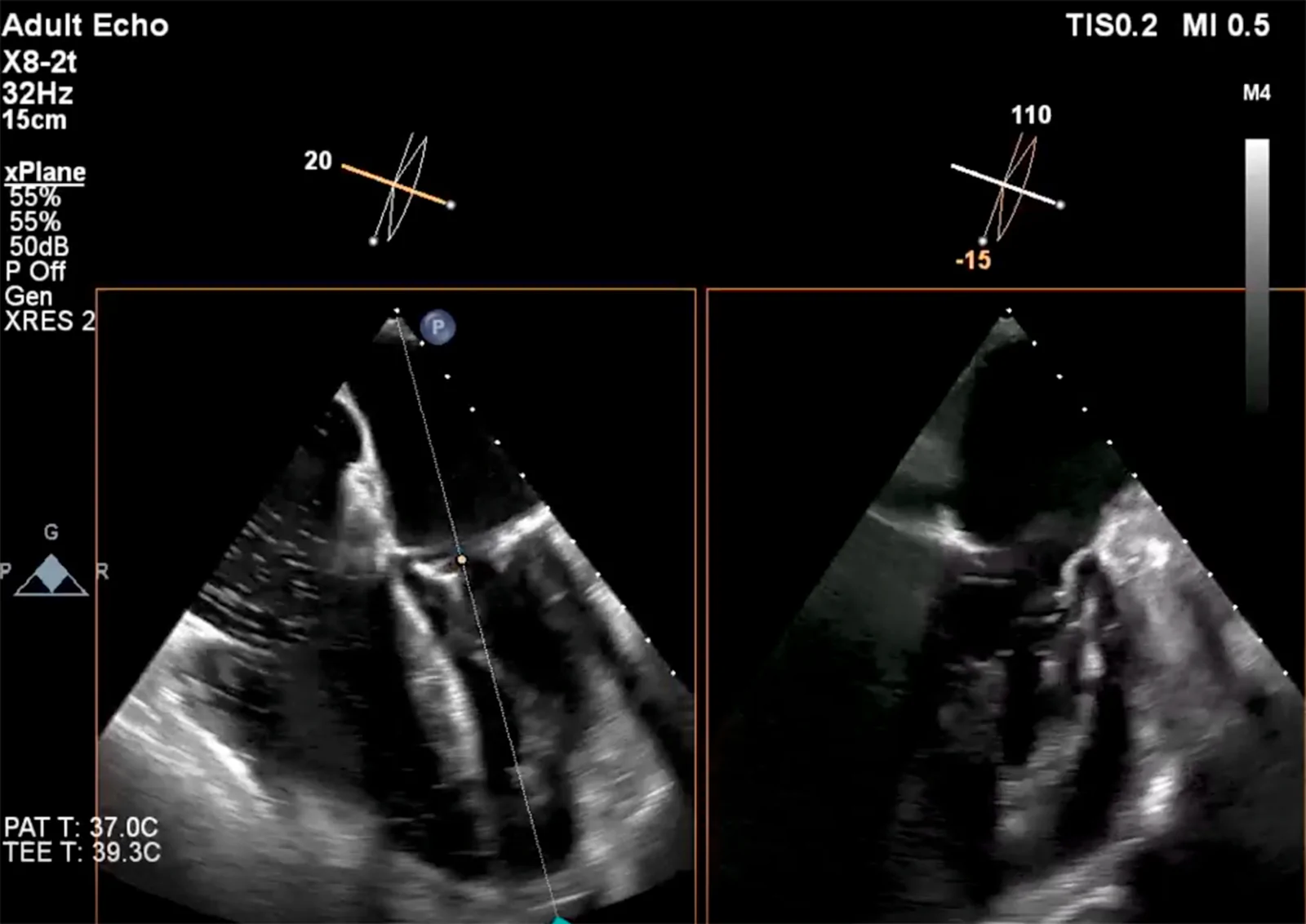
X-Plane of Midesophageal 4-Chamber on Transesophageal Echocardiography Demonstrating an Underfilled Left Ventricle and Overloaded Right Ventricle

A 79-year-old man presented with acute dyspnea. Transthoracic echocardiography demonstrated critical aortic stenosis (aortic valve area: 0.49 cm^2^, mean gradient: 32 mm Hg, peak velocity: 3.5 m/s, dimensionless index: 0.17) (Figure 1, Videos 1 and 2) with preserved left ventricular (LV) ejection fraction (55%-60%). Severe right ventricular (RV) dysfunction was present at baseline, characterized by reduced tricuspid annular plane systolic excursion (1.1 cm), decreased RV systolic velocity (6.4 cm/s), and severely reduced fractional area change (21%), along with RV dilation (RV basal diameter: 4.7 cm, RV mid-diameter: 4.4 cm) (Figure 2). Cardiac catheterization found 85% stenosis of the mid left anterior descending artery and chronic total occlusion of the right coronary artery (RCA) with left-to-right collaterals (Videos 3 and 4). The cardiac surgery team determined he was at prohibitive surgical risk given his comorbidities (below). He was referred for transcatheter aortic valve replacement (TAVR). After heart team discussion, TAVR was performed, with planned staged percutaneous coronary intervention. Via transfemoral access, a 26-mm Sapien 3 Ultra Resilia bioprosthesis (Edwards Lifesciences) was successfully deployed under rapid pacing (Figure 3, Video 5), with reduction in the mean transaortic gradient from 41 to 6 mm Hg. He developed ventricular fibrillation following valve deployment, requiring defibrillation ×2 with conversion to sinus rhythm, and no change in hemodynamics. The large-bore TAVR sheath was removed, followed by the routine administration of protamine for heparin reversal (40 mg intravenous over 4 minutes). Immediately after protamine administration, the patient developed acute hemodynamic deterioration characterized by severe hypotension, decreased pulsatility, and hypoxia, with acute worsening of RV function, concerning for protamine type III reaction (Figure 4, Video 6).

**Take-Home Messages**

• Type III protamine reaction represents a rare but catastrophic complication in patients with RV dysfunction undergoing TAVR.
• Patients with preexisting RV dysfunction may be particularly susceptible to serious complications from protamine reaction because of limited physiologic reserve.
• Early initiation of RV support with Impella RP should be considered in patients with acute RV failure secondary to protamine reaction.

## Past Medical History

The patient's history was notable for heart failure with preserved ejection fraction, chronic atrial fibrillation, end-stage kidney disease on hemodialysis, type 2 diabetes mellitus, and recurrent infections of his dialysis port.

## Differential Diagnosis and Investigations

A broad differential was rapidly assessed. Re-engagement of the left main artery ruled out ostial coronary obstruction. Transesophageal echocardiography (TEE) ruled out cardiac tamponade from annular rupture or guidewire perforation as well as ascending aortic dissection. The TAVR valve was well positioned without evidence of dysfunction. TEE also demonstrated preserved LV systolic function with no wall motion abnormalities, and telemetry showed no ST-segment changes, making ostial coronary obstruction unlikely Notably, TEE identified severely depressed RV systolic function with a flattened interventricular septum, consistent with acute RV pressure overload. Protamine reaction was suspected given the temporal relationship of administration and absence of other plausible causes.

## Management

**Figure 5:**
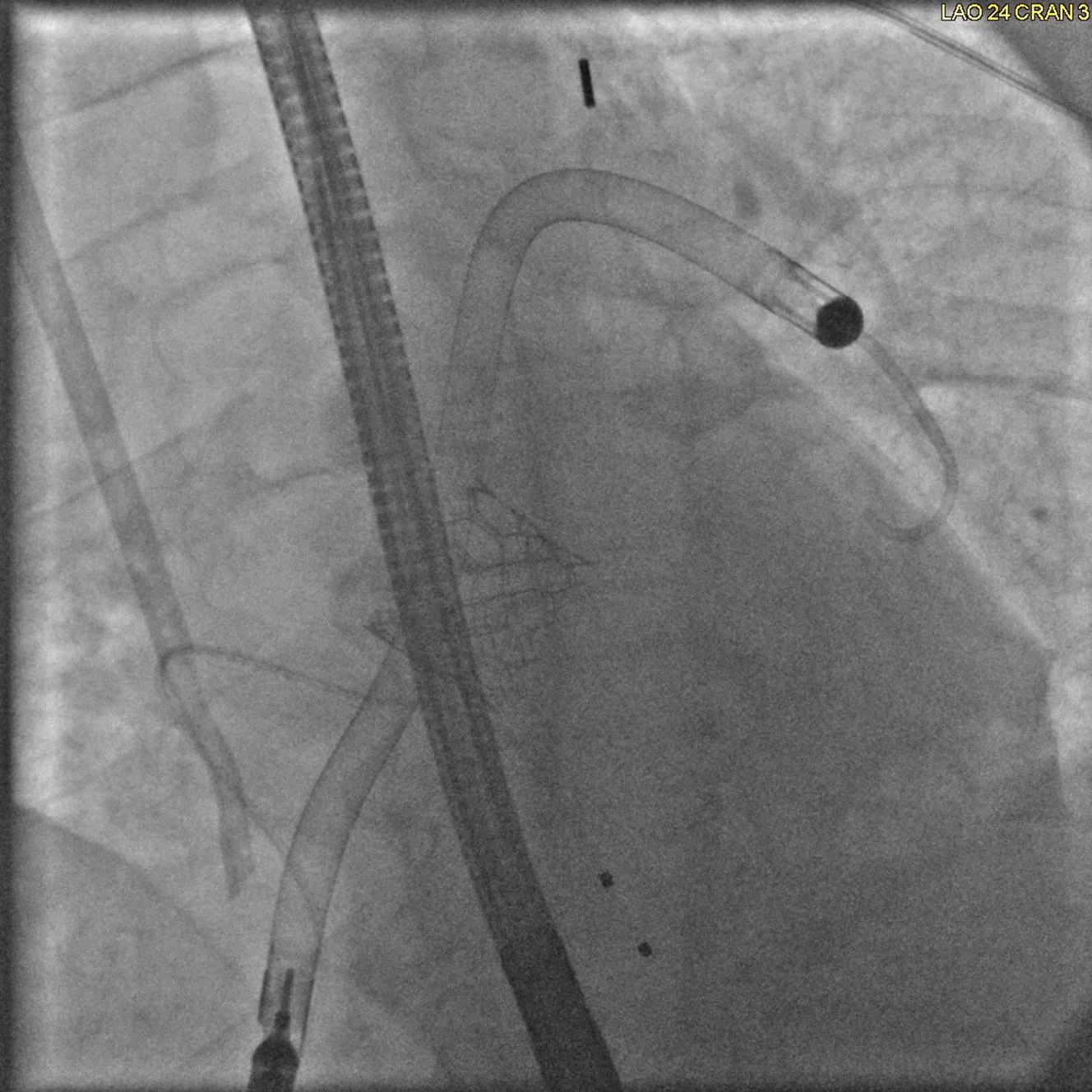
Fluoroscopy Demonstrating Appropriate Placement of Impella RP in the Vicinity of the Left Upper Pulmonary Artery and Appropriate Placement of the Intra-Aortic Balloon Pump in the Thoracic Descending Aorta The temporary right ventricular pacing wire and the recently deployed 26-mm Sapien 3 Ultra Resilia bioprosthetic valve is also visualized.

The patient developed profound hypotension (systolic blood pressure in the 40s) refractory to vasopressors, necessitating chest compressions and epinephrine administration. Continuous inotropic support with dobutamine and epinephrine was initiated to augment RV contractility. An intra-aortic balloon pump (IABP) was inserted to augment diastolic perfusion pressure across the severely stenotic mid left anterior descending artery and to support left-to-right collateral perfusion to the chronically occluded RCA territory. Inhaled nitric oxide was initiated for selective pulmonary vasodilation to reduce RV afterload without exacerbating systemic hypotension. Despite these measures, the patient remained in refractory shock, with a persistently elevated central venous pressure (CVP) of 31 mm Hg and an underfilled LV cavity, consistent with right-sided cardiogenic shock. A right ventricular microaxial flow pump (Impella RP Flex; Johnson & Johnson) was inserted via the right femoral vein across the pulmonic valve into the right pulmonary artery (Figure 5, Video 7). After Impella RP activation, CVP decreased from 31 to 20 mm Hg, with immediate improvement in RV contractility, size, interventricular septal flattening, hypoxia, and vasopressor requirements (Videos 8 and 9).

## Outcome and Follow-Up

Postprocedural transthoracic echocardiography confirmed a well-seated bioprosthetic aortic valve with no significant gradient or paravalvular aortic regurgitation and preserved LV ejection fraction of 55% to 60%. RV systolic function remained severely depressed. The patient required ongoing IABP and Impella RP support. The patient's course in the intensive care unit was complicated by acute renal failure requiring initiation of continuous renal replacement therapy, shock liver, acidemia with elevated lactate levels, gastrointestinal hemorrhage, and IABP-related limb ischemia. The patient was deemed ineligible for advanced therapies including durable LV assist device or transplantation given his age, end-stage kidney disease, and critical limb ischemia. Despite maximal pharmacologic and mechanical circulatory support, he remained in Impella RP device-dependent right-sided cardiogenic shock and passed away on postoperative day 4 secondary to cardiac arrest with pulseless electrical activity.

## Discussion

This case illustrates a severe type III protamine reaction characterized by acute pulmonary vasoconstriction leading to abrupt RV pressure overload and cardiogenic shock. Type III reactions are mediated by complement activation, mast cell degranulation, and thromboxane release, resulting in a sudden increase in pulmonary vascular resistance. In susceptible patients, this can precipitate acute RV failure, reduced LV preload, and circulatory collapse. Although protamine-induced adverse reactions are well-described in cardiac surgery, their occurrence in TAVR and other structural interventions is under-recognized and probably under-reported, particularly given the growing evidence supporting routine protamine use for heparin reversal in transfemoral TAVR.^1^^,^^2^ This case demonstrates that timely escalation to percutaneous mechanical circulatory support with the Impella RP, even in patients without surgical or extracorporeal membrane oxygenation options, can achieve meaningful hemodynamic stabilization in refractory protamine-induced RV failure.

Protamine reactions are classified into 3 types: type I involves mild hypotension; type II manifests as anaphylactic or anaphylactoid features; and type III, the most hemodynamically devastating, is characterized by acute pulmonary vasoconstriction and consequent RV failure.^3^^,^^4^ Mechanisms include complement activation, mast cell degranulation, and thromboxane-mediated pulmonary vasoconstriction.^3^ In our center, we routinely administer protamine after all structural heart procedures. Notably, protamine doses in TAVR are substantially lower than in cardiopulmonary bypass—approximately 30 to 50 mg versus doses often exceeding 200 mg in cardiopulmonary bypass^1^^,^^2^^,^^4^—suggesting the hemodynamic collapse in our patient reflected an idiosyncratic immune-mediated mechanism rather than a dose-dependent effect, and indicating that no threshold dose is safe in a susceptible individual. This patient represented a particularly high-risk phenotype because of baseline severe RV dysfunction, chronic pressure overload from severe aortic stenosis, dialysis-associated volume loading, and complete RCA occlusion with reliance on left-to-right collateral circulation. In this setting, any acute increase in pulmonary vascular resistance could be poorly tolerated and could rapidly lead to hemodynamic collapse. The acute rise in CVP to 31 mm Hg with preserved LV function, hyperdynamic underfilled LV, and severe RV dysfunction on TEE suggested a protamine-mediated type III reaction producing acute RV pressure overload.

Pharmacological rescue targeted pulmonary vascular resistance reduction and RV contractility augmentation. Inhaled nitric oxide was initiated as the primary pulmonary vasodilator given its selectivity, rapid onset, and absence of systemic hypotension, with established utility in acute right heart syndromes.^5^^,^^6^ Dobutamine and epinephrine provided RV inotropic support. An IABP was deployed not for conventional LV support, as LV systolic function was preserved, but to augment diastolic perfusion to the left coronary system, which supplied the entire myocardium given the RCA chronic total occlusion; without this, the compromised RV would have faced progressive ischemic injury. When hemodynamic instability persisted despite maximal pharmacological support, escalation to percutaneous RV support was undertaken.

The Impella RP was inserted to directly unload the failing RV, drawing blood from the right atrium and delivering it to the pulmonary artery.^7^ Following activation, CVP fell from 31 to 20 mm Hg, RV contractility and size improved on TEE, interventricular septal flattening resolved, hypoxia improved, and vasopressor requirements decreased; confirming effective hemodynamic stabilization. The RECOVER RIGHT trial reported 30-day survival of 73% in patients with refractory RV failure^7^; however, real-world registries report survival of only approximately 53%, reflecting the broader heterogeneity and comorbidity burden of this population.^8^ The fatal outcome in this patient is consistent with these data, given his severe baseline RV dysfunction, absence of a viable revascularization pathway, and the downstream consequences of prolonged mechanical circulatory support dependency including device-related hemolysis, limb ischemia, and multiorgan failure.

This case adds to a sparse literature on protamine-induced RV failure in the TAVR context. To our knowledge, this represents one of the few reported cases of protamine-induced type III reaction requiring Impella RP support after TAVR. Although routine protamine use after TAVR is supported at a population level by meta-analytic data,^1^^,^^2^ the risk-benefit calculus differs for patients with severe RV dysfunction, pulmonary hypertension, hemodialysis dependence, or extreme ischemic vulnerability. Identifying patients predisposed to type III reactions is a clinical priority. Protamine test dosing, slower infusion rates, and TEE monitoring are possible strategies to consider in high-risk patients. Additionally, arterial rather than venous administration may attenuate pulmonary hemodynamic perturbations, postulated to occur via hepatic first-pass metabolism, reducing pulmonary vasoconstrictive exposure. A prospective observational study of radial arterial protamine administration in cardiac surgical patients demonstrated stable pulmonary artery pressures and no RV dysfunction, supporting this as a translatable mitigation strategy in high-risk structural heart patients.^4^^,^^9^ Although it may not be appropriate to categorically withhold protamine in all cases, in carefully selected high-risk patients it is reasonable to administer a reduced dose or omit reversal entirely and allow heparin to wear off under activated clotting time guidance. Early availability of inhaled nitric oxide, intravenous epoprostenol,^10^ pre-emptive percutaneous RV support planning, and multidisciplinary cardiac critical care involvement may improve the rescue window when protamine reactions occur.

## Conclusions

Protamine-induced type III reaction causing acute RV failure represents a rare but catastrophic complication of TAVR, particularly in patients with preexisting RV dysfunction, pulmonary hypertension, or collateral-dependent coronary anatomy. Protamine-sparing strategies should be prospectively considered in such high-risk individuals. When acute RV failure occurs, Impella RP can achieve meaningful hemodynamic stabilization as a bridge to recovery, although cumulative comorbidity burden may often limit survival despite immediate hemodynamic stabilization.

## Funding Support and Author Disclosures

Dr Dohad has received research funding from Boston Scientific, Abiomed, and Penumbra, and consulting fees from Philips. Dr Makkar has received research grants and travel support from Edwards Lifesciences, Boston Scientific, Medtronic, Abbott Vascular, and JenaValve. Dr Chakravarty has received consulting fees from Edwards Lifesciences and Boston Scientific. All other authors have reported that they have no relationships relevant to the contents of this paper to disclose.

## Appendix

Video 1 Parasternal Long-Axis View on TTE Demonstrating Restricted Aortic Valve Leaflet Opening Consistent With Severe Aortic Stenosis

Video 2 Fluoroscopy Demonstrating Severe Stenosis of the Mid Left Anterior Descending Artery, With Left-to-Right Collaterals

Video 3 Fluoroscopy Demonstrating Chronic Total Occlusion of the Right Coronary Artery

Video 4 Transgastric Short-Axis Aortic Valve-Focused View on TEE Demonstrating Severely Restricted Aortic Valve Opening Consistent With Severe Aortic Stenosis

Video 5 Fluoroscopy Demonstrating Deployment of the 26-mm Sapien 3 Ultra Resilia Bioprosthetic Valve Under Rapid Pacing at Nominal Volume Minus 2 mL of Contrast

Video 6 Midesophageal 4-Chamber View With X-Plane on TEE Demonstrating Underfilled Left Ventricle and Severely Dilated Right Ventricle With Interventricular Septal Flattening, Consistent With Right Ventricular Pressure and Volume Overload

Video 7 Fluoroscopy Demonstrating Appropriate Placement of Impella RP in the Vicinity of the Left Upper Pulmonary Artery and Appropriate Placement of the Intra-Aortic Balloon Pump in the Thoracic Descending Aorta The temporary RV pacing wire and the recently deployed 26-mm Sapien 3 Ultra Resilia bioprosthetic valve is also visualized.

Video 8 Midesophageal 4-Chamber View on TEE Demonstrating Improved RV Contractility After Placement of Impella RP

Video 9 Transgastric 4-Chamber View on TEE Demonstrating Restoration of RV Contractility After Placement of Impella RP
